# Psychiatric Diagnoses in Prehospital Emergency Care and Sociodemographic Characteristics of the Incident Location at the District Level

**DOI:** 10.1017/S1049023X25101325

**Published:** 2025-08

**Authors:** Valesca Sophie Deutsch, Yacin Keller, Jochen Hardt, Katja Petrowski

**Affiliations:** 1. Center for Quality Assurance and Development, Johannes Gutenberg University, Mainz, Germany; 2. City of Dresden Fire Department, Integrated Regional Control Center, Dresden, Germany; 3. Medical Psychology and Medical Sociology, University Medical Center of the Johannes Gutenberg University, Mainz, Germany; 4. Department of Internal Medicine III, University Medical Center Carl Gustav Carus at the University of Dresden

**Keywords:** emergency medicine, psychological stress, residence characteristics, urban health

## Abstract

**Background:**

The aim of this study was to analyze the prevalence of psychiatric symptoms in prehospital emergency care and the characteristics of this patient group as well as the association with deprivation in the district, self-assessment of health status, and the frequency of emergency calls due to or accompanied by psychiatric diagnoses.

**Methods:**

A retrospective cross-sectional study descriptively and analytically evaluated all ground-based Emergency Medical Service and rescue service incidents dispatched by the Integrated Regional Control Center (IRLS) in the period from January 1, 2021 through December 31, 2021. In addition to the clinical parameters and the demographic data of the patients, the sociodemographic characteristics of the incident location at the district level, unemployment rate, net equivalent household income, and the proportion of single-person households, as well as personal assessment of mental health and overall well-being, were included in the study.

**Results:**

A total of 68,345 deployment protocols were examined. Of these, 6.4% were emergency incidents due to or involving psychiatric diagnoses. Emergency physician (EP) involvement in these operations was 56.1%. RM Andersen’s Behavioral Model of Health Services Use (1968) was used as a theoretical reference model for the description, analysis, and explanation of the use of health-related care. The analyses showed that interventions due to or involving psychiatric diagnoses without emergency doctor alerts were more frequent in urban districts with a high proportion of single-person households and a high net equivalized houshold income.

**Conclusion:**

The accumulation in individual city districts and the factors identified by Andersen point to opportunities to target preventive measures to avoid emergencies involving psychiatric diagnoses in order to use limited resources efficiently.

## Introduction

Data on the utilization of health care services are collected through various population-representative surveys, such as the “Study on the Health of Adults in Germany (DEGS1)” by the Robert Koch Institute (RKI; Berlin, Germany).^1^ This study has been collecting nation-wide data on the health of adults living in Germany since 2008 as part of the RKI’s health monitoring.^2^ According to DEGS1 data, women significantly more frequently utilize medical services than men, a difference that decreases with age. Overall, utilization increases with age. A significant influence is the self-assessment of the general health status. Study participants who rated their health as fair to very poor utilized out-patient medical care more often than those with very good or good health. Similar results were found for in-patient care: the self-assessment of health as fair to very poor was highly associated with a higher frequency of treatment.^1^

The relationship between the utilization of health care services and psychiatric comorbidity has also been well-documented in numerous studies. Patients with psychiatric disorders show increased utilization of out-patient and in-patient medical services.^3–6^ Epidemiological studies also demonstrate that psychiatric disorders are wide-spread in the German population. Through the “Mental Health” (DEGS1-MH) module, prevalence estimates of psychiatric disorders among adults aged 18 to 79 were made in the study. The 12-month prevalence here was 27.7%.^7^ Due to the high prevalence and significant disease burden of psychiatric disorders, data on mental health in the population are of extraordinary importance. Since 2019, the RKI has been developing a Mental Health Surveillance for Germany, aiming at the “continuous reporting of relevant indicators for the purpose of evidence-based planning and evaluation of public health measures.”^8^ Through a structured consensus process, an indicator set has been developed that represents a public health framework for adult mental health in various action fields. These indicators include pre-clinical symptoms, selected psychiatric disorders, comorbidity, as well as the utilization and quality of care services.^8^

Patients with psychiatric symptoms are frequently encountered in prehospital emergency medicine.^9^ Emergency departments often serve as contact points for patients with psychiatric comorbidities or primary psychiatric disorders during acute crises.^10^ Treating this patient group demands extensive personnel, spatial, and diagnostic resources and poses a particular workload on medical staff.^11^ In Germany, and in many other countries, a continuous increase in annual Emergency Medical Service incidents can also be observed.^12–16^ At the same time, the proportion of non-life-threatening complaints is rising.^14,17,18^

Knowledge about patients with psychiatric symptoms in Emergency Medical Services, their pre-clinical care, and characteristics is still limited. Given the high proportion of ambulatory treated emergency patients and the rising number of annual Emergency Medical Service and rescue service incidents, the emergency care of this patient group by Emergency Medical Service structures should be analyzed specifically.^19^ This retrospective cross-sectional study evaluated all ground-based Emergency Medical Service and rescue service incidents in the City of Dresden, dispatched by the Integrated Regional Control Center (IRLS) Dresden, in the period from January 1, 2021 through December 31, 2021, to draw conclusions about the care needs of patients with psychiatric symptoms. In addition to the demographic data and clinical parameters of the patients, sociodemographic characteristics of the incident location at the district level, unemployment rate, household net equivalent income, and the proportion of single-person households, as well as personal assessment of medical care, mental health, and overall well-being, were included in the study. The aim of the study was to analyze the prevalence of psychiatric symptoms in prehospital emergency care, the characteristics of this patient group, as well as the association with deprivation in the district, self-assessment of health status, and the frequency of emergency calls due to or accompanied by psychiatric diagnoses.

## Conceptual Framework – Andersen’s Behavioral Model of Health Services Use

As one of the leading reference models for describing, analyzing, and explaining the utilization of health-related services, RM Andersen’s Behavioral Model of Health Services Use (1968) is applied. The model has been developed and used in numerous studies with different research designs and in the context of specific settings, populations, and diseases.^20–24^ At its core, the comprehensive multi-stage model distinguishes between need factors at both contextual and individual levels, pre-disposing characteristics, and enabling resources as influences on the utilization of health services.^25,26^

### Need Factors

Andersen distinguishes between a perceived need for health services and a professionally evaluated need. The perceived need depends primarily on how people perceive their own state of health or assess the perceived symptoms of illness and is thus associated with patient-induced utilization.^23,26,27^ The evaluated need, on the other hand, describes a need “objectified by professional judgment,”^26^ determines the scope of the health care services provided, and is associated with physician-induced utilization.^23,26,27^

### Pre-Disposing Characteristics

According to Andersen, pre-disposing characteristics have an indirect effect on utilization and include characteristics in the areas of demographics, social structure, and health beliefs. Demographic characteristics include age and gender, which act as “biological imperatives” on utilization.^27^ In addition to education, occupation, and social status, social structure characteristics include ethnicity and social relationships or networks.^27^ The term social structure is thus used to summarize characteristics whose composition and characteristics can be used to derive which means and resources are available to a person in the decision-making process to solve (health) problems.^26^

Pre-disposing characteristics also include so-called health beliefs. These describe health beliefs, for example, a person’s attitudes, values, and knowledge about health or the health care system. These characteristics shape health behavior and thus the decision-making process of utilization, as they have a significant influence on the individual, subjective perception of need, and the cost/ benefit assessment of utilization.^26,27^

### Enabling Resources

Enabling resources can be understood as facilitating factors or necessary pre-requisites for the utilization of health care services. At this point, Andersen distinguishes between personal resources (personal/family), such as income and the existence of health insurance, and community-related resources (community). The latter refer to the accessibility of care facilities in the vicinity of the place of residence and the place of work and thus the density of doctors and hospitals, but also the composition of service providers and health education programs. In contrast to the indirect pre-disposing characteristics, personal enabling resources only describe resources that have a direct effect on the use of health care services.^23,26,27^

**Figure 1.**
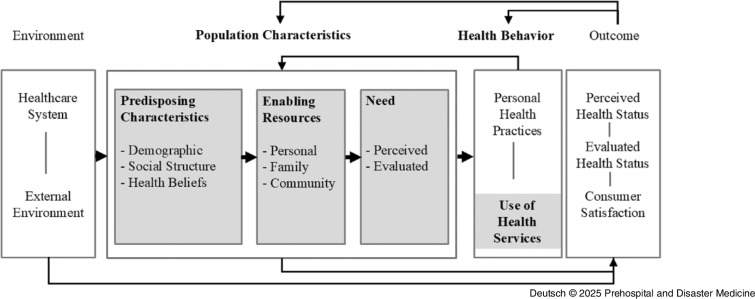
Andersen’s Behavioral Model of Health Services Use 1995.^22^

Figure 1 shows the schematic representation of the behavioral model for the use of health care services as of 1995. In addition to the described model core, the model was supplemented by the component’s outcomes and environment. Outcomes describe both the subjective (perceived) and objective (evaluated) state of health, as well as the satisfaction with care, and can be understood as the results of the utilization of health care services.^26^ The model describes the overall social context as the environment and thus also includes the corresponding (country-specific) health care system in which utilization is embedded.^26^ On the one hand, the model emphasizes the diverse influences on the use of health care services and, as a consequence, on the state of health. On the other hand, the feedback loops used make it clear that the results of utilization in turn have an influence on the pre-disposing factors, the individually perceived need, and health behavior.^22^

Through the theoretical-conceptual differentiation of pre-disposing characteristics, enabling factors, and need factors within the behavioral model, inequalities in access to health care services can be systematically represented and empirically mapped.^25^ This article therefore examines the prevalence of psychological symptoms in prehospital emergency medicine and the characteristics of this patient group. In addition, the various factors influencing the use of emergency services by this vulnerable patient group are described and analyzed with the help of Andersen’s Behavioral Model of Health Services Use.^22^

With reference to this, the personal assessment of one’s own mental health and overall well-being were examined as need factors, age, gender, and household size as pre-disposing characteristics and net equivalized household income, as well as the assessment of medical care as enabling resources and their significance for the frequency of use of the emergency services. The aim of the work is to specifically analyze the care of patients with mental symptoms to an evidence-based foundation for needs-based patient care.

## Methods

As part of a retrospective cross-sectional study, all rescue service operations of the ground-based emergency medical and rescue service of the City of Dresden, which were dispatched in the IRLS Dresden in the period from January 1, 2021 through December 31, 2021, were evaluated. As one of a total of five large control centers in the Saxony control center network, the IRLS’s area of responsibility covers the state capital of Dresden, the district of Saxon Switzerland-Eastern Ore Mountains, and the district of Meissen, covering an area of 3,434 km^2^ with approximately 1.1 million inhabitants.^28^

Included were all ground-based emergency interventions involving the treatment of only one emergency patient aged 18 or over by an ambulance or an emergency ambulance vehicle or an ambulance together with an emergency ambulance vehicle. Deployments involving the care of more than one patient, patients less than 18 years of age, more than two ambulances or emergency ambulances, rescue helicopter deployments, and deployments of emergency physicians (EPs) from other rescue service areas as well as fire department deployments were excluded. Only the emergency doctor deployment logs were evaluated. The analyzed protocols correspond to the standard of the German Interdisciplinary Association for Intensive Care and Emergency Medicine (DIVI) and serve the legally correct documentation of prehospital emergency rescue by rescue and Emergency Medical Services.^29^ The following were recorded: the gender and age of the emergency patients concerned, the treatment time at the scene (time between arrival and departure from the scene), the emergency keyword reported by the rescue coordination center, the underlying main diagnosis, the vital parameters (systolic blood pressure in mmHg, heart rate in beats/minute, blood sugar in mmol/l, SpO2 in %, body temperature in °C, respiratory rate in breaths/minute, and pain intensity according to the visual analog scale and the Glasgow Coma Scale) at the start and end of the operation, as well as the emergency medical invasive and medicinal measures carried out, the National Advisory Committee for Aeronautics/NACA score, and the type of transport.

Emergency physician protocols were evaluated for psychiatric or behavioral disorders as per the “International Statistical Classification of Diseases and Related Health Problems” (ICD-10), defining psychiatric disorders under Chapter V (F00-F99).^30^ A psychiatric disorder was assumed if an F-diagnosis was recorded as the main diagnosis. Additionally, protocols were analyzed for specific disorders: depression (F3), anxiety disorders (F40, F41, F48), somatoform disorders (F45), dissociative disorders (F44), reaction to severe stress and adjustment disorders (F43), obsessive-compulsive disorders (F42), and schizophrenia (F2). Diagnoses R45.-, T42.-, and Z91.- were also analyzed to detect possible suicidality or suicide attempts.

**Figure 2.**
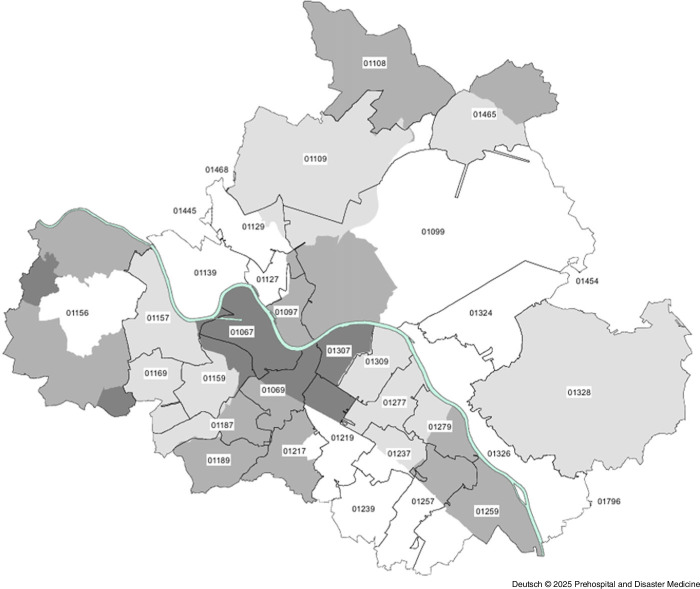
Overview of the Postcode Areas.^32^Note: Colored areas are the underlying City Districts as in Figure 3.

**Figure 3.**
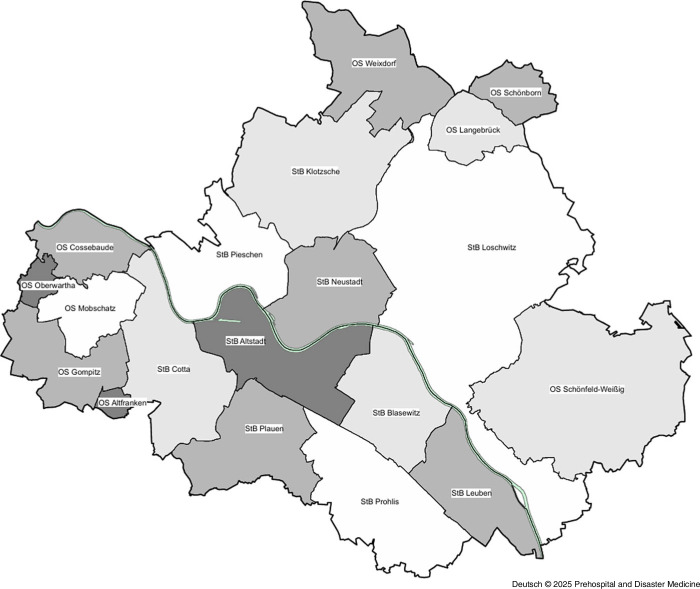
Overview of Dresden City Districts (Street Directory 32).

As Dresden’s urban area has been divided hierarchically into city districts and boroughs for statistical purposes since 1992, it seemed obvious to use this division as the basis for the present analysis.^31,32^ For this purpose, the available zip codes of the emergency operations were first assigned to the corresponding city districts and then summarized to the next largest city districts (Figure 2 and Figure 3). Since a city district can extend over several zip code areas, some of the emergency operations with different zip codes were assigned to one city district. The zip codes were then assigned to a city district if more than 80% of the deployments were in this zip code area. This made it possible to link the socio-structural data at borough level^31,33^ with the available emergency response protocols. Socio-structural data were available from the 2020 district catalog for the following districts: Altstadt, Blasewitz, Cotta, Klotsche, Leuben, Loschwitz, Neustadt, Pieschen, Plauen, and Prohlis.

Emergency protocols were anonymized and assigned specific incident numbers, preventing direct identification of individuals during analysis. The study was ethically approved by the State Medical Association of Rhineland-Palatinate (application number 2020-15330).

### Statistical Analysis

To analyze the use of the ambulance service with and without an EP, the type of deployment (ie, pure ambulance deployments, primary emergency physician deployments [pEP], and deployments with EP call-outs) were defined as primary target variables. The presence of an F-diagnosis as the main diagnosis was used as a primary response variable. In addition, age, gender, and selected socio-economic characteristics of the deployment location, as well as the personal assessment of one’s own mental health and well-being and medical care, were included in the analysis as possible mediators or moderating influencing variables.

The significance level was set at α = 0.01. P values were corrected according to Bonferroni due to multiple testing in cases with and without EPs. Hence, the raw alpha was set to 0.01 : 2 = 0.005 for the regression analysis (primary outcomes). For no secondary outcome was any P value reported; these outcomes are described descriptively and considered exploratory. Due to a strong age effect, linear and quadratic terms for age were included in the regression models. The data were recorded in Microsoft Excel (Microsoft Corp.; Redmond, Washington USA). Statistical analysis was performed using STATA (V16; StataCorp; College Station, Texas USA)^34^ for Windows and the statistical software R (version R3.5.3; R Foundation for Statistical Computing; Vienna, Austria).^35^ The sample characteristics were analyzed using mean values, standard deviations (SD), and frequencies. Scientific notation was used to display exact P values.

## Results

A total of 68,345 deployment protocols were included in the study. Of these, 47,885 (70.1%) were primary ambulance call-outs and 19,568 (28.6%) were primary EP call-outs. In 892 (1.2%) cases, an EP was called by the non-medical first responder. Only the protocols of the respective incident commander were evaluated here. Deployments with an EP for which no physician deployment log existed were excluded, as no documentation was provided by the incident commander. At least one mental or behavioral disorder (ICD10 codes F00-F99) was recorded as the main diagnosis in 4,371 (6.4%) of the deployment protocols included in the study. The majority (52.6%) of the primary ambulance operation was with an EP. An overview can be found in Table 1.

**Table 1.** Proportion of Psychiatric Cases and Primary Ambulance Operations

	N	%
Total Cases	68,345	100.0
Of Which Psychiatric Cases	4,371	6.4
Of Which Primary EP Deployments	2,300	52.6
Primary Operations Without EP	1,977	45.2
EP Call-Outs	892	2.2

Abbreviation: EP, Emergency Physician.

### Primary Disposition with Emergency Physician

As part of the analysis, the rescue service operations were evaluated separately according to their primary dispatch with and without an EP. The average age was 48.4 (SD = 21.5) years for patients with a psychiatric diagnosis and 67.5 (SD = 20.9) years for patients without a psychiatric diagnosis. Just under one-quarter (22.5%) of all patients with a psychiatric diagnosis were under 30 years old. In addition, patients with a psychiatric diagnosis were predominantly male (51.5%), while patients without a psychiatric diagnosis were predominantly female (51.0%). Among the 2,300 identified primary EP deployments in which at least one mental or behavioral disorder was recorded as the main diagnosis, the most frequent deployments were in the diagnosis group: *mental and behavioral disorders caused by psychotropic substances* (F10-F19) (n = 719; 31.3%); followed by *neurotic, stress, and somatoform disorders* (F40-F48) (n = 601; 26.1%), and *schizophrenia, schizotypal, and delusional disorders* (F20-F29) (n = 378; 16.4%). The following distribution was found within the diagnosis groups for the disorders examined: *depression* (F3) (n = 281; 12.2%); *anxiety disorders* (F40, F41, F48) (n = 317; 13.8%); *somatoform disorders* (F45) (n = 217; 9.4%); *dissociative disorders* (F44) (n = 31; 1.4%); *reaction to severe stress and adjustment disorders* (F43) (n = 50; 2.2%); *obsessive-compulsive disorders* (F42) (n = 0; 0.0%); and *schizophrenia* (F2) (n = 378; 16.4%). In addition, the protocols were analyzed with regard to the diagnoses R45.-; T42.-, and Z91.- in order to be able to record possible suicidal tendencies or suicide attempts. This was the case in 926 (4.7%) of the EP protocols examined. The exact distribution can be seen in Table 2. For the treatment year 2020, there were no peaks in presentations in terms of month, day of the week, or time of day.

**Table 2.** Percentage of Psychiatric Diagnoses (ICD-10) by Diagnosis and by Primary Ambulance Operation

ICD-10-Diagnosis	Total (%)	Primary Operation **With EP** N (%)	Primary Operation **Without EP** N (%)	EP Call-Out N (%)
**Total Cases**	4,371 (100.0)	2,300 (52.6)	1,977 (45.2)	94 (2.2)
**F00-09** Organic, Including Symptomatic, Mental Disorder	228 (5.2)	149 (6.5)	74 (3.7)	5 (5.3)
**F10-19** Mental and Behavioral Disorders due to Psychoactive Substance Use	1,834 (42.0)	719 (31.3)	1,075 (54.4)	40 (42.6)
**F20-29** Schizophrenia, Schizotypal, and Delusional Disorders	517 (11.8)	378 (16.4)	132 (6.7)	7 (7.5)
**F30-39** Mood (Affective) Disorders	443 (10.1)	281 (12.2)	158 (8.0)	4 (4.3)
**F40-48** Neurotic, Stress-Related and Somatoform Disorder	1,032 (26.6)	601 (26.1)	401 (20.3)	30 (31.9)
**F50-59** Behavioral Syndromes Associated with Physiological Disturbances and Physical Factors	15 (0.3)	5 (0.2)	9 (0.5)	1 (1.1)
**F60-69** Disorders of Adult Personality and Behavior	64 (1.5)	48 (2.1)	16 (0.8)	0 (0.0)
**F70-79** Mental Retardation	4 (0.1)	3 (0.1)	1 (0.05)	0 (0.0)
**F80-89** Disorders of Psychological Development	2 (0.05)	1 (0.04)	1 (0.05)	0 (0.0)
**F90-98** Behavioral and Emotional Disorders with Onset Usually Occurring in Childhood and Adolescence	447 (10.2)	243 (10.6)	188 (9.5)	11 (11.7)

**Figure 4.**
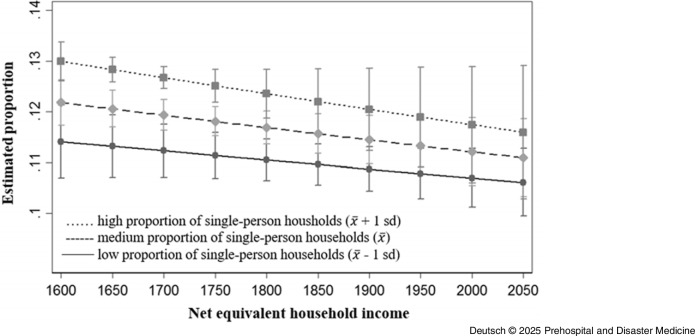
Proportion of Operations with Primary EP Call-Outs and Documented F-Diagnosis, Taking into Account the Net Equivalent Household Income and the Proportion of Single-Person Households at District Level.Abbreviation: EP, Emergency Physician.

Based on the statistical data of the city of Dresden,^31,32^ the available zip codes of the emergency interventions were assigned to the corresponding districts. There were substantial differences in the prevalence of interventions involving psychiatric diagnoses across districts. Such interventions were more frequent in districts Altstadt (0.84%), Blasewitz (0.80%), Neustadt (0.95%), and Prohlis (0.99%) compared to the remaining districts, where prevalence ranged from 0.30% to 0.65%. The results of the logistic regressions showed that the proportion of single-person households in the district had no significant influence on the frequency of emergency interventions. Nevertheless, districts with high unemployment and a high proportion of single-person households had higher number of emergency interventions due to or involving psychiatric diagnoses than districts with low unemployment and a low proportion of single-person households. In addition, the net equivalent household income, which was available for the districts of Altstadt, Blasewitz, Cotta, Klotsche, Leuben, Loschwitz, Neustadt, Pieschen, Plauen, and Prohlis from the 2020 Municipal Citizen Survey, was included in further analysis. In the main effect, the logistic regression showed no significant association with net equivalent household income and the frequency of emergency interventions in the respective district. In the test of interaction, the logistic regression showed no significant differences between districts with a high net equivalized household income and a high proportion of single-person households and districts with a low net equivalized household income and a low proportion of single-person households with regard to the proportion of emergency interventions with coding of mental health diagnoses (Figure 4 and Table 3). Also included in the evaluation were the survey results from the Municipal Citizen Survey 2020 for the above-mentioned districts on the personal assessment of the state of health and the personal assessment of mental health in particular. The assumption that the proportion of emergency interventions with coding of mental diagnoses was higher in districts in which people tend to rate their own state of health and mental health as reasonably satisfactory or poor than in districts in which people rate their own state of health or mental health as good could not be confirmed on the basis of the available data.

**Table 3.** Logistic Regression for Proportion of Operations with Primary Emergency Physician Call-Outs and Documented F-Diagnosis, Taking into Account the Net Equivalent Household Income and the Proportion of Single-Person Households at District Level

	Coef.	**SE**_**β**_	z	P> |z|	Bonf.
**Income**	.0002686	.0007024	0.38	0.702	/
**Single**	.0295348	.0237656	1.24	0.214	/
**Income#Single**	−9.96e-06	.0000137	−0.73	0.467	/
**Constant**	−1.281549	1.234827	−1.04	0.299	/

Note: N = 177,895; Pseudo R^2^ = 0.119; Bonf. = Bonferroni corrected p; * indicates p≤ .01; ** indicates p≤ .001; *** indicates p≤ .0001; Income = Net equivalent household income; Single = Proportion of single-person households.

### Primary Disposition without Emergency Physician

**Figure 5.**
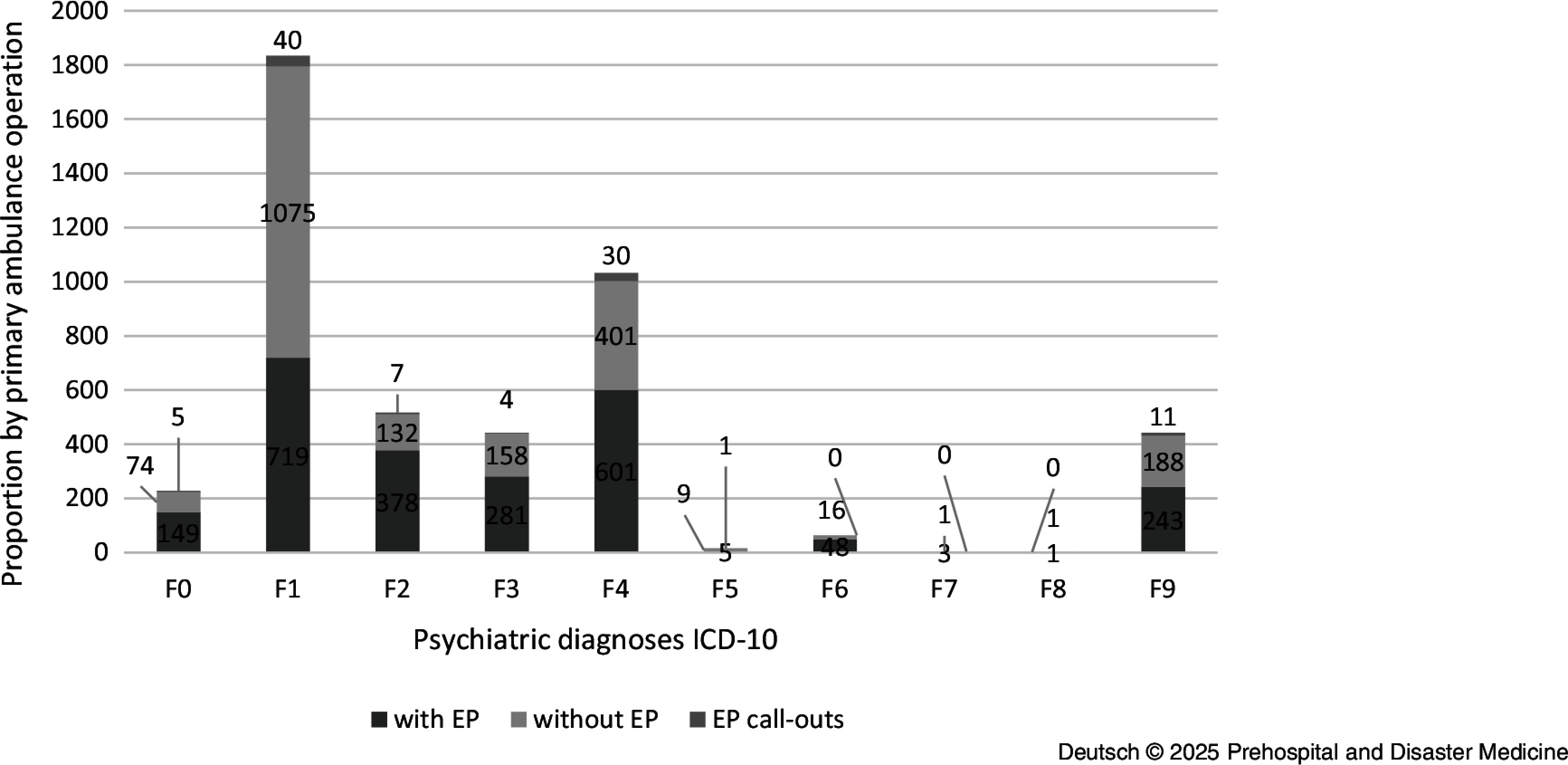
Proportion of Psychiatric Diagnoses (ICD-10) In Total and by Primary Ambulance Operation.Abbreviation: ICD-10, International Statistical Classification of Diseases.

With regard to the demographic characteristics of the patient collective, there were no substantial differences for age and marginal differences for gender between the ambulance deployments with and without an EP. In the case of primary ambulance operations without an EP, the patients were also predominantly male (58.6%) and on average 47.2 years old (SD = 19.6). The ambulance deployments without an EP were significantly more frequently attributable to the diagnostic groups *mental and behavioral disorders caused by psychotropic substances* (F10-F19) (n = 1,075; 54.4%), followed by *neurotic, stress, and somatoform disorders* (F40-F48) (n = 401; 20.3%). In contrast to the emergency medical interventions, the third most common diagnoses here were from the spectrum of *behavioral and emotional disorders with onset in childhood and adolescence* (F90-F98) (n = 188; 9.5%); Figure 5.

**Figure 6.**
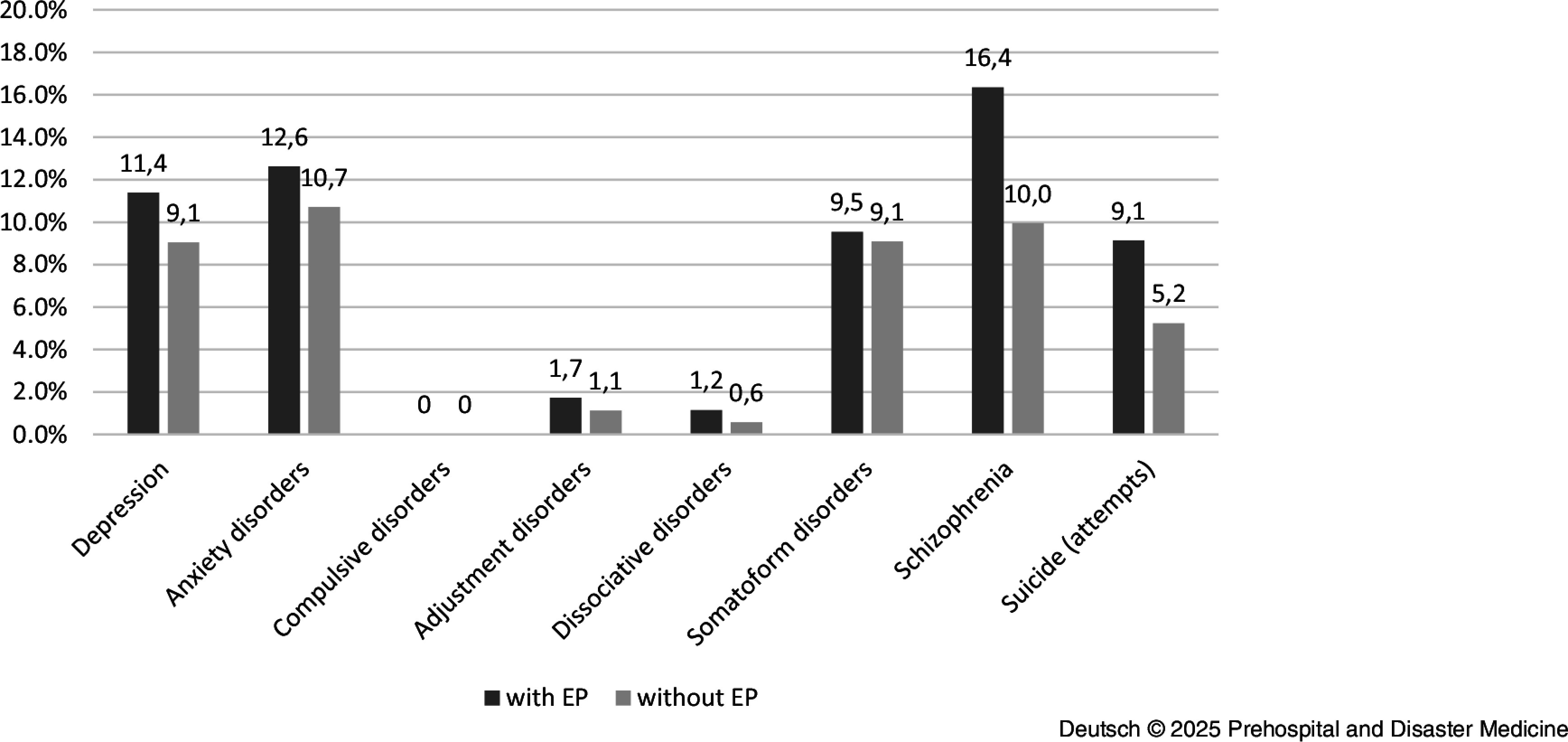
Proportion of Selected Disorders in Ambulance Call-Outs With and Without EP.Abbreviation: EP, Emergency Physician.

**Figure 7.**
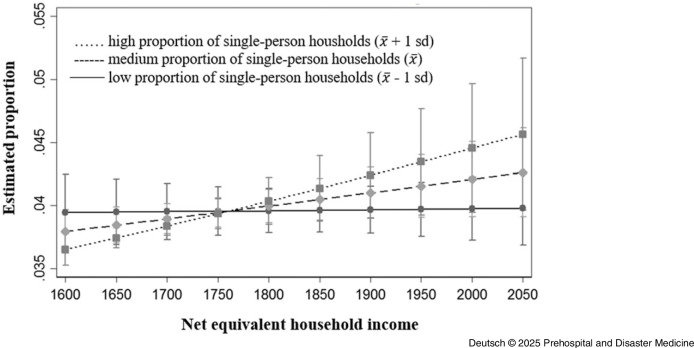
Proportion of Operations Without EP and Documented F-Diagnosis, Taking into Account the Net Equivalent Household Income and the Proportion of Single-Person Households at District Level.Abbreviation: EP, Emergency Physician.

Substantial differences in the prevalence of interventions involving psychiatric diagnoses were also observed across districts. Such interventions occurred more frequently in Altstadt (0.73%), Neustadt (0.83%), and Prohlis (0.65%) compared to other districts, where the prevalence ranged from 0.21% to 0.53%. Additionally, there were differences with regard to the disorders examined; in particular, the diagnoses of schizophrenia, suicide, and attempted suicide accounted for significantly fewer interventions. Figure 6 provides an overview of the proportion of selected disorders in ambulance call-outs with and without EPs. The results of the logistic regression show that the proportion of single-person households had a significant influence on the frequency of emergency interventions (without EP). The interaction test showed that in districts with a high net equivalized household income and a high proportion of single-person households, the proportion of emergency interventions with coding of mental diagnoses was higher than in districts with a low net equivalized household income and a low proportion of single-person households (Figure 7 and Table 4).

**Table 4.** Logistic Regression for Proportion of Operations Without Emergency Physician and Documented F-Diagnosis, Taking into Account the Net Equivalent Household Income and the Proportion of Single-Person Households at District Level

	Coef.	**SE**_**β**_	z	P> |z|	Bonf.
**Income**	− .0019993	.0007023	−2.85	0.004	*
**Single**	− .0756675	.0237567	−3.19	0.001	**
**Income#Single**	.000043	.0000137	3.14	0.002	*
**Constant**	.9360005	1.236965	0.76	0.449	/

Note: N = 543,115; Pseudo R^2^ = 0.0919; Bonf. = Bonferroni corrected p; * indicates p≤ .01; **indicates p≤ .001; ***indicates p≤ .0001; Income = Net equivalent household income; Single = Proportion of single-person households.

## Discussion

The aim of this study was to analyze the prevalence of mental symptoms in prehospital emergency care and the characteristics of this patient group, as well as the association with deprivation in the city district and the frequency of interventions due to or involving psychiatric diagnoses. In addition to clinical parameters and demographic data, the study also included socio-demographic characteristics of the location at borough level, the unemployment rate, net equivalent household income, and the proportion of single-person households, as well as personal assessments of medical care, mental health, and overall well-being. A total of 68,345 deployment protocols were examined. Of these, 6.4% were emergency incidents due to or involving psychiatric diagnoses. Emergency physician involvement in these operations was 56.1%.

RM Andersen’s Behavioral Model of Health Services Use (1968) was used as a theoretical reference model for the description, analysis, and explanation of the use of health-related care. Personal assessment of mental health and overall well-being were considered as need factors at the individual level; age, gender, and household size as pre-disposing characteristics; and household net equivalent income, unemployment rate, and assessment of medical care as enabling resources and were examined as possible mediators or moderating factors influencing the frequency of emergency interventions due to or involving psychiatric diagnoses. The analyses showed that interventions (without emergency doctor alert) due to or involving psychiatric diagnoses were more frequent in urban districts with a high proportion of single-person households and high net equivalized houshold income. In contrast, net equivalized household income had no significant influence on the frequency of interventions with emergency doctor alert in the district. In addition, the patients were predominantly male and, on average, younger than the patients with interventions not involving psychiatric diagnoses. It was not possible to confirm an association with an assessment of the patient’s own state of health, and in particular mental health, as reasonably satisfactory or poor and a higher number of emergency interventions with coding of psychiatric diagnoses.

Various preventive measures and options for improving current patient care can be derived from this. For example, targeted training and prevention measures in schools and youth facilities would be conceivable. Faster availability and low-threshold access to out-patient support services, for example in the form of a social psychiatric (crisis) service or home treatment teams, could also contribute to better care and relieve the burden on the emergency medical treatment system.^36^ Furthermore, initial assessment and care are usually carried out by doctors who do not primarily work in psychiatry. In order to ensure adequate care and avoid incorrect referrals, not only is knowledge of the diagnostic and therapeutic procedure necessary, but also knowledge of the legal basis for treatment.^36^ As already suggested by Schöttle and colleagues, targeted training measures, such as work shadowing in psychiatry and interdisciplinary further training, might improve both the care of patients with acute mental illnesses and the situation of the treating physicians.^36^

In summary, a differentiated picture emerges. The variables interpreted as pre-disposing characteristics – age, gender, and the proportion of single-person households – were positively associated with the proportion of emergency interventions in which mental health diagnoses were coded. In contrast, the variables interpreted as enabling resources were only positively associated with the unemployment rate in interventions with emergency doctor alert. No significant association could be shown for the need factors. Further investigations should focus on whether the patients in question were treated as in-patients or out-patients. Critical questions should then be asked as to whether a significant proportion of in-patient admissions could have been avoided by offering appropriate out-patient help. The results also indicate that the patients with psychiatric diagnoses were younger. It would be interesting for further studies to look at the proportion of patients under the age of 18.

## Limitations

When interpreting the results, it must be considered that the location does not necessarily correspond to the patient’s place of residence, particularly when assigning socio-structural data at the district level and location via zip code. Furthermore, the retrospective study design does not allow any conclusions to be drawn about the severity of the main diagnoses recorded. The documented main diagnoses represent an initial suspected diagnosis and may therefore deviate from the final diagnosis. The analysis of the primary outcome indicated strong interaction between income and proportion of single households on the frequency of emergency interventions without emergency doctor alert, while no such interaction was present in interventions with emergency doctor alert. Secondary outcomes were prevalence differences in districts and differences in age and gender. Results related to the secondary outcomes should be interpreted with caution and regarded as exploratory. These findings are hypothesis-generating and require confirmation in future prospective studies.

## Conclusion

This study analyzed a large data set using one of the leading theoretical reference models for describing and explaining the utilization of health-related services: Andersen’s Behavioral Model of Health Services Use. Therefore, it provides insights into the characteristics of a vulnerable, resource-intensive population. The results show that interventions due to or involving psychiatric diagnoses without emergency doctor alert were more frequent in urban districts with a high proportion of single-person households and high net equivalized houshold income. The accumulation in individual city districts and the factors identified by Andersen point to opportunities to target preventive measures to avoid emergencies with F-diagnoses in order to use limited resources efficiently.
